# Analysis of Chronic Tinnitus in Noise-Induced Hearing Loss and Presbycusis

**DOI:** 10.3390/jcm10081779

**Published:** 2021-04-19

**Authors:** Hee Jin Kang, Dae Woong Kang, Sung Su Kim, Tong In Oh, Sang Hoon Kim, Seung Geun Yeo

**Affiliations:** 1 Department of Otolaryngology-Head & Neck Surgery, School of Medicine, Kyung Hee University, Seoul 02447, Korea; gmlwlskkk@naver.com (H.J.K.); kkang814@naver.com (D.W.K.); hoon0700@naver.com (S.H.K.); 2 Medical Research Center for Bioreaction to Reactive Oxygen Species and Biomedical Science Institute, School of Medicine, Graduate School, Kyung Hee University, Seoul 02447, Korea; sgskim@khu.ac.kr; 3 Department of Biomedical Engineering, College of Medicine, Kyung Hee University, Seoul 02447, Korea; tioh@khu.ac.kr

**Keywords:** tinnitus, noise-induced hearing loss, presbycusis, audiology

## Abstract

**Introduction:** The most frequent causes of tinnitus associated with hearing loss are noise-induced hearing loss and presbycusis. The mechanism of tinnitus is not yet clear, although several hypotheses have been suggested. Therefore, we aimed to analyze characteristics of chronic tinnitus between noise-induced hearing loss and presbycusis. **Materials and Methods:** This paper is a retrospective chart review and outpatient clinic-based study of 248 patients with chronic tinnitus from 2015 to 2020 with noise-induced or presbycusis. Pure tone audiometry (PTA), auditory brainstem response (ABR), distortion product otoacoustic emissions (DPOAE), transient evoked otoacoustic emissions (TEOAE), and tinnitograms were conducted. **Results:** PTA showed that hearing thresholds at all frequencies were higher in patients with noise-induced hearing loss than the presbycusis group. ABR tests showed that patients with presbycusis had longer wave I and III latencies (*p* < 0.05 each) than patients with noise-induced hearing loss. TEOAE tests showed lower values in patients with noise-induced hearing loss than presbycusis at 1.5, 2, 3, and 4 kHz (*p* < 0.05 each). DPOAE tests showed that response rates in both ears at 1.5, 2, and 3 kHz were significantly higher in patients with presbycusis than noise-induced hearing loss (*p* < 0.05 each). **Discussion:** This study showed that hearing thresholds were higher, the loudness of tinnitus was smaller, and the degree of damage to outer hair cells was lower in patients with presbycusis than with noise-induced hearing loss. Moreover, wave I and III latencies were more prolonged in patients with presbycusis despite their having lower hearing thresholds. These phenomena may reflect the effects of aging or degeneration of the central nervous system with age. Further studies are needed to evaluate the etiologies of tinnitus.

## 1. Introduction

Tinnitus is one of the most common otologic diseases with a condition in which an individual recognizes sounds in the absence of external sound stimulation [1]. There are several subtypes of tinnitus such as conductive tinnitus, sensorineural hearing loss, and vascular tinnitus. Conductive tinnitus can occur because of middle ear origins such as ear infections, tympanic membrane and ossicular chain problems, glomic tumors, myoclonus, and tonic tensor tympani syndrome. Sensorineural tinnitus is accompanied by sensorineural hearing loss, which is the most common type of tinnitus. It can be associated with presbycusis, metabolic problems such as diabetes mellitus, hypothyroidism, dyslipidemia, anemia, vitamin and mineral deficiencies, and noise exposure. Vascular tinnitus can be produced by the turbulence of blood flow transmitted to the cochlea [2,3,4]. The most frequent causes of tinnitus associated with hearing loss are noise-induced hearing loss and presbycusis [5]. The mechanism of tinnitus can be explained based on nerve-fiber activity in the temporal lobe of the cortex—the same as the perception of all sound. The activity can be caused by several mechanisms. The spontaneous activity of neurons in the auditory system included deafferentation and central changes as well as an increase in cross-fiber correlation. The case of tinnitus due to hearing loss can be explained by the cochlea origin, but the central origin can not be ignored [6,7]. According to the diverse mechanisms of tinnitus, previous studies emphasized the importance of subgrouping tinnitus patients for treatment with a preliminary cluster analysis. Therefore, these different approaches of treatment of tinnitus can represent a fundamental difference in the neural mechanisms [8].

However, in some situations, a single hypothesis cannot accurately explain the cause of tinnitus. Moreover, although tinnitus affects large numbers of people and reduces their quality of life, evidence-based, multidisciplinary clinical practice guidelines are not yet clear [1]. Despite hearing loss being the most common cause of objective tinnitus, not enough studies to date have assessed whether variations in the characteristics of tinnitus are dependent on the type of hearing loss [4,6,7]. Therefore, we aimed to analyze and compare characteristics of chronic tinnitus between noise-induced hearing loss and presbycusis.

## 2. Materials and Methods

### 2.1. Subjects

This study consisted of a retrospective chart review of patients with chronic tinnitus who visited Kyunghee Medical Center from 2015 to 2020 with noise-induced or presbycusis. The chronic tinnitus was defined as duration >3 months. Patients with noise-induced hearing loss were defined as those with a history of exposure to noise, such as workers in the mining and machinery industries. The hearing threshold was ≥25 dB, as calculated using the six-division method ((500 Hz + 2 × 1 kHz + 2 × 2 kHz + 4 kHz)/6) on PTA [9,10]. Patients with presbycusis were defined as those aged ≥65 years without a history of noise exposure, with a hearing threshold of ≥25 dB as calculated using the six-division method on PTA. Patients were excluded if test results were insufficient; if their duration of acute tinnitus was <3 months; or if they had auditory nerve tumors, brain disease, cancer, trauma history, or other systemic diseases. Patients aged ≥65 years with noise-induced hearing loss were also excluded.

All patients with tinnitus were evaluated by tests of pure tone audiometry (PTA), auditory brainstem response (ABR), distortion product otoacoustic emissions (DPOAE), and transient evoked otoacoustic emissions (TEOAE). Tinnitograms were performed to get the pitch and loudness of tinnitus. In all frequencies, we selected threshold and added another 10 dBHL and presented pure tone or noise, according to the patients’ description on the characteristics of their tinnitus. We requested the patient to answer when realizing that the sound presented was similar to their tinnitus, and in the frequency indicated by the patient as similar to their tinnitus, the stimulus was presented noise, with an initial intensity of 10 dBHL below the patient’s threshold. We increased the intensity in steps of 2 dBHL, and the patients realized that the intensity was similar to the presented tinnitus [11,12]. The results of these tests were compared to those of patients with noise-induced hearing loss and presbycusis.

### 2.2. Statistical Analysis

Sound-to-noise ratios (SNRs) on TEOAE tests were compared in the two groups using *t*-tests, and response rates on tests of DPOAE were compared using the MEDCALC program [13]. All other results in the two groups of patients were compared using Mann–Whitney U-tests. Statistical analyses were performed using SPSS version 20.0 software, with *p*-values < 0.05 considered statistically significant.

## 3. Results

**Figure 1:**
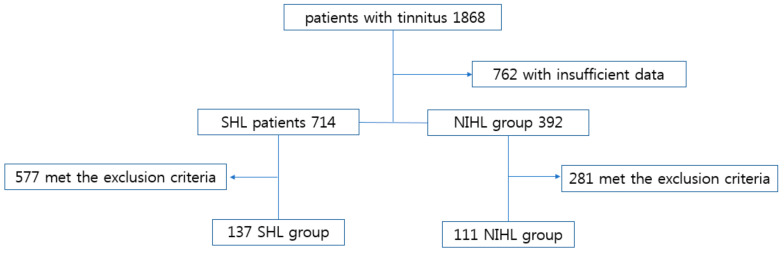
Study flow diagram. Patients with tinnitus were classified into those with SHL and NIHL. Exclusion criteria included insufficient data or a normal hearing threshold. Abbreviations: SHL, presbycusis; NIHL, noise-induced hearing loss.

Of the 1868 patients with tinnitus who visited the hospital during the study period, 137 with presbycusis and 111 with noise-induced hearing loss were included in the study (Figure 1). There were no significant differences between these two groups in age, sex, and rates of diabetes and hypertension (*p* > 0.05 each). Concomitant symptoms, such as vertigo, autophonia, and ear fullness, were more frequent in patients with presbycusis, but the differences were not statistically significant (*p* > 0.05 each) (Table 1).

PTA tests showed that hearing thresholds at all frequencies were significantly higher in patients with noise-induced than with presbycusis (*p* < 0.05) (Table 2). The mean frequency of tinnitus was lower in patients with presbycusis than with noise-induced hearing loss, but this difference was not statistically significant (*p* > 0.05). Loudness was significantly greater in patients with noise-induced than with presbycusis (*p* < 0.05) (Table 3).

ABR tests showed that wave I and wave III latencies were significantly longer in patients with presbycusis than with noise-induced hearing loss (*p* < 0.05 each). Wave V latency also tended to be longer in patients with presbycusis, but the difference was not statistically significant (*p* > 0.05). In addition, I-III IPL and III-V IPL were significantly longer in patients with presbycusis than with noise-induced hearing loss (*p* < 0.05 each) (Table 4).

TEOAE tests showed lower values at 1.5, 2, 3, and 4 kHz in patients with noise-induced than with presbycusis (*p* < 0.05 each) (Table 5). On DPOAE tests, the response rate tended to decrease as the frequency increased, as well as to be higher in patients with presbycusis than with noise-induced hearing loss. In particular, response rates in both ears at 1.5, 2, and 3 kHz were significantly higher in patients with presbycusis than with noise-induced hearing loss (*p* < 0.05 each) (Table 6).

## 4. Discussion

Many studies have evaluated the mechanisms, causes, and treatments of tinnitus. However, the evaluation of tinnitus through audiological examination is still limited in its ability to fully characterize the tinnitus, and there are many areas that should be studied in the future [14]. The basic tests currently used to diagnose tinnitus include PTA, ABR, tinnitus pitch and loudness tests, the TEOAE test, and the DPOAE test. PTA can help determine the cause of tinnitus by assessing the presence or absence of hearing loss. Given that the choice of therapeutic strategy is dependent on the hearing loss status, it is very important to analyze the degree and pattern of hearing loss. The basic tests are also important because they can be used to confirm to the patient that their tinnitus is real, to monitor changes as treatment progresses, to provide insights into mechanisms, and/or to assist in the fitting of devices, such as a hearing aid or sound generator [15,16,17,18,19]. In addition, the characteristics of the tinnitus can be assessed objectively through tests evaluating tinnitus pitch and loudness. This can be very useful not only for diagnostic purposes, but for treatment, such as the selection of an appropriate range for sound therapy. ABR tests provide objective information to determine whether the auditory nerve has been damaged and to evaluate whether a vestibular neuron or other abnormalities are present in the peripheral auditory nerve pathway. Otoacoustic emission (OAE) tests are useful for estimating the origin of tinnitus because they reflect the status of the outer hair cells of the cochlea. OAE tests may also be used in treatment to explain the mechanism of tinnitus to the patient by determining whether or not external hair cells are overexcited or damaged. In the present study, these auditory tests showed differences in tinnitus between patients with noise-induced hearing loss and presbycusis. In addition, because noise exposure among young adults and presbycusis in the elderly are the most common causes of tinnitus, it is meaningful that patients in our study were grouped according to these etiologies [1,20,21,22].

The PTA tests in our study found that hearing thresholds at all frequencies were lower in patients with presbycusis than with noise-induced hearing loss. In general, patients with presbycusis showed a downward pattern toward the high-frequency region, whereas patients with noise-induced hearing loss showed a downward pattern in both directions around 4 kHz. In addition, the loudness of tinnitus was significantly lower in patients with presbycusis than with noise-induced hearing loss. Patients with noise-induced hearing loss in the study reported more severe hearing loss and more severe subjective tinnitus than patients with presbycusis. It showed the same results as previous studies [10].

Tinnitus pitch did not differ significantly in the two groups of patients. Moreover, the frequency of the pitch did not match the worst hearing threshold in either group. Similar results have been observed in previous studies, and one study found that both the pitch and loudness of tinnitus in the two groups of patients were lower than in this study. In that study, tinnitus pitch and loudness were 3 kHz and 45 dB, respectively, in patients with presbycusis and 4 kHz and 40 dB, respectively, in patients with noise-induced hearing loss [23]. Additional studies are needed to resolve these discrepancies [11,24,25,26].

ABR tests showed that wave I, III, and V latencies were slightly longer in patients with presbycusis and noise-induced hearing loss than in normal subjects. Moreover, wave I and wave III latencies were significantly longer in patients with presbycusis than with noise-induced hearing loss. Since the results of ABR tests are dependent on the lesion site, these findings seem to be related to differences in the pathogenesis of hearing loss and tinnitus in the two groups. Although these findings are consistent with previous results, no consensus has been reached on the magnitudes of these differences [19]. ABR has been found to detect cochlear synaptopathy early in patients with noise-induced hearing loss, as shown by reduced wave I amplitudes in patients exposed to noise in previous studies [21]. In general, more severe hearing loss is thought to be associated with more prolonged latency on ABR. However, our findings were different than expected. The causes of tinnitus include those of cochlear origin and central, or neurotransmitter-associated, origin. The results of the present study suggest that the aging and degeneration of the central nervous system had an effect on tinnitus in patients with presbycusis. Further studies are necessary to determine whether different results on ABR tests are dependent on the etiology of hearing loss.

OAE tests can evaluate the degree of damage to the outer hair cells, with the DPOAE tests being better able to select higher frequencies than the TEOAE tests [27]. In this study, there was no specific trend for each frequency in the TEOAE tests. In contrast, the DPOAE tests showed that the response rate decreased as the frequency increased in both groups of patients. This was consistent with the PTA pattern. In addition, the pitch of tinnitus was found to average 4973 Hz in patients with noise-induced hearing loss and 5636 Hz in patients with presbycusis. However, DPOAE tests showed that the pitch was closer to 4 kHz in patients with presbycusis. However, because tinnitus is caused not only by damage to the outer hair cells but by a central response, OAE alone may be limited in determining the characteristics of tinnitus [23]. On DPOAE, the response rates at 1.5, 2, 3, and 4 kHz were significantly lower in patients with noise-induced than with presbycusis. A previous study in patients with noise-induced hearing loss found that response rates at 2 and 3 kHz were significant in distinguishing these patients from individuals with normal hearing [21]. Thus, the ability of OAE to contribute to the diagnosis of tinnitus should be evaluated by determining whether frequencies differ by causes of tinnitus.

In conclusion, this study showed that hearing thresholds were higher, the loudness of tinnitus was smaller, and the degree of damage to outer hair cells was lower in patients with presbycusis than with noise-induced hearing loss. Moreover, wave I and III latencies were more prolonged in patients with presbycusis, despite their having lower hearing thresholds. These phenomena may reflect the effects of aging or degeneration of the central nervous system with age. Further studies are needed to evaluate the etiologies of tinnitus.

## Figures and Tables

**Table 1:** Demographic characteristics of patients with noise-induced and presbycusis.

Characteristics	Presbycusis (*n* = 137)	Noise-Induced Hearing Loss (*n* = 111)	*p*-Value
Age, yr,	69.53	60.86	0.055
Gender	M:F = 49:88	M:F = 111:0	0.061
Laterality	Rt:Lt = 100:97	Rt:Lt = 54:73	0.14
Duration of tinnitus, yr	3.55	16.41	<0.05
Diabetes mellitus	21.8% (30/137)	18.01% (20/111)	0.12
Hypertension	43.06% (59/137)	31.5% (35/111)	0.07
Vertigo	20.4% (28/137)	9.9% (11/111)	0.055
Autophonia	12.4% (17/137)	9.0% (10/111)	0.062
Ear fullness	14.5% (20/137)	11.7% (13/111)	0.85

**Table 2:** Pure tone audiometry in patients with presbycusis and noise-induced hearing loss.

Hz	Presbycusis (*n* = 137)		Noise-Induced Hearing Loss (*n* = 111)		*p*-Value	
	Rt	Lt	Rt	Lt	Rt	Lt
125 Hz	23.86 ± 10.89	30.94 ± 18.87	40.27 ± 13.00	46.55 ± 16.09	<0.01	<0.01
250 Hz	24.74 ± 12.44	29.05 ± 18.71	42.29 ± 15.75	43.33 ± 16.73	<0.01	<0.01
500 Hz	24.03 ± 13.32	29.45 ± 19.23	42.79 ± 16.51	44.59 ± 17.17	<0.01	<0.01
1000 Hz	29.37 ± 13.92	33.28 ± 19.41	48.87 ± 15.97	49.50 ± 16.08	<0.01	<0.01
2000 Hz	32.99 ± 14.95	36.71 ± 18.33	58.28 ± 15.33	59.63 ± 15.01	<0.01	<0.01
3000 Hz	36.67 ± 16.50	42.51 ± 18.54	67.25 ± 14.67	68.55 ± 14.80	<0.01	<0.01
4000 Hz	43.72 ± 17.43	50.21 ± 18.62	72.79 ± 14.00	74.54 ± 14.18	<0.01	<0.01
8000 Hz	62.51 ± 18.25	63.79 ± 15.30	78.91 ± 12.38	78.78 ± 12.95	<0.01	<0.01
Total	32.12 ± 12.77	36.61 ± 17.17	54.98 ± 14.09	56.23 ± 14.02	<0.01	<0.01

Results are reported as mean ± SD.

**Table 3:** Mean frequency and loudness of tinnitus in patients with presbycusis and noise-induced hearing loss.

	Presbycusis (*n* = 137)	Noise-Induced Hearing Loss (*n* = 111)	*p*-Value
Frequency (kHz)	4973.61 ± 3410.03	5636.74 ± 2907.82	0.233
Loudness (dB)	56.33 ± 19.75	71.94 ± 17.61	<0.01

Results are reported as mean ± SD.

**Table 4:** ABR results in patients with presbycusis and noise-induced hearing loss.

	Presbycusis (*n* = 137)		Noise-Induced Hearing Loss (*n* = 111)		*p*-Value	
	Right	Left	Right	Left	Right	Left
I latency	1.67 ± 0.17	1.67 ± 0.44	1.52 ± 0.47	1.55 ± 0.18	<0.01	<0.01
III latency	3.86 ± 0.20	3.81 ± 0.61	3.74 ± 0.38	3.71 ± 1.07	0.02	0.01
V latency	5.78 ± 0.24	5.84 ± 0.28	5.68 ± 1.22	5.65 ± 1.55	0.74	0.123
I–III IPL	2.18 ± 0.14	2.12 ± 0.40	2.06 ± 1.77	2.24 ± 0.67	<0.01	<0.01
III–V IPL	1.92 ± 0.14	1.86 ± 0.32	1.90 ± 0.72	1.96 ± 0.16	<0.01	0.023
I–V IPL	4.10 ± 0.18	3.98 ± 0.74	4.16 ± 0.73	4.27 ± 0.21	0.49	<0.01

Results are reported as mean ± SD, IPL, interpeak latency.

**Table 5:** TEOAE results showing signal-to-noise ratios in each ear of tinnitus patients with presbycusis and noise-induced hearing loss.

Hz	SNR				*p*-Value	
	Presbycusis (*n* = 137)		Noise-Induced Hearing Loss (*n* = 111)			
	Right	Left	Right	Left	Rt	Lt
1	1.45 ± 0.84	1.21 ± 9.79	−1.08 ± 1.12	0.06 ± 8.03	0.072	0.39
1.5	7.7 ± 9.75	7.89 ± 10.99	3.48 ± 9.71	3.11 ± 10.21	0.003	0.002
2	7.5 ± 10.16	6.66 ± 9.31	1.25 ± 9.24	2.32 ± 7.89	<0.01	0.001
3	2.82 ± 8.39	1.5 ± 8.39	−2.98 ± 7.07	−2.21 ± 6.72	<0.01	0.003
4	−0.71 ± 7.68	−0.97 ± 7.73	−3.46 ± 6.14	−4.12 ± 5.34	0.022	0.007

Results are reported as mean ± SD. SNR, signal-to-noise ratio.

**Table 6:** DPOAE results showing response rates in each ear of tinnitus patients with presbycusis and noise-induced hearing loss.

kHz	Response Rate ^a (%)^				*p*-Value	
	Presbycusis (*n* = 137)		Noise-Induced Hearing Loss (*n* = 111)			
	Right	Left	Right	Left	Right	Left
1	43.06	39.41	29.09	36.3	0.02	0.6
1.5	64.23	57.66	28.18	26.36	*p* < 0.01	*p* < 0.01
2	35.03	26.27	11.81	14.54	*p* < 0.01	0.02
3	22.62	21.67	8.18	10	*p* < 0.01	0.01
4	14.59	13.13	5.45	8.18	0.02	0.01

^a^ Response rates were calculated by dividing the number patients with normal responses by the total number of patients with tinnitus and multiplying by 100 (%).

## Data Availability

The data are available from authors by a reasonable request.
